# A Paraneoplastic Leukemoid Reaction in Primary Lung Sarcoma

**DOI:** 10.7759/cureus.15047

**Published:** 2021-05-15

**Authors:** Ali F Al Sbihi, Nouraldeen Manasrah, Farah M Al Haj, Sarah Al Qasem, Joel Appel

**Affiliations:** 1 Internal Medicine, DMC (Detroit Medical Center) Sinai-Grace Hospital, Detroit, USA; 2 Internal Medicine, Wayne State University Detroit Medical Center, Detroit, USA; 3 Emergency Department, Luzmila Hospital, Amman, JOR

**Keywords:** leukemoid reaction, paraneoplastic syndromes, lung sarcoma, lung cancer, pulmonary sarcoma

## Abstract

Pulmonary malignancies are known to have high prevalence and mortality. They are associated with different paraneoplastic syndromes, especially pulmonary carcinomas, because they are more common than pulmonary sarcomas. We present a case of a 56-year-old African American male who was admitted to our institution with a three-month history of a dry cough, progressive shortness of breath, and two to three days of right arm swelling. He had extreme leukocytosis (WBC count of 106,500 cells/mm^3^). Computed tomography (CT) scan of the thorax demonstrated an irregular, thick-walled 14-cm lung mass occupying the middle and upper hemithorax. CT-guided biopsy of the mass confirmed the diagnosis of lung sarcoma.

## Introduction

Lung cancers are characterized by high incidence, prevalence, and mortality. It is known that lung carcinoma is the most common type of lung cancer. Epithelial subtypes of lung cancer are the most encountered ones [1]. Primary pulmonary sarcomas (PPSs) are an exceedingly rare, aggressive, and diverse group of non-epithelial malignant tumors of the mesenchymal tissue of the bronchial wall, vessels, or pulmonary stroma origin. They comprise only 0.013%-1.1% of all malignant lung tumors [2,3]. It has been estimated that the lung carcinoma:sarcoma ratio is around 500:1 [4].

Pulmonary malignancies are associated with many paraneoplastic syndromes. Because lung carcinomas are more common than sarcomas, their paraneoplastic syndromes are also more common and tend to be well-described in the literature, especially with small cell lung carcinoma [2]. When leukocytosis exists as a white blood cell (WBC) count of more than 50,000 cells/mm^3^ of mature, non-clonally derived neutrophils, which is associated with a malignancy, it is defined as a paraneoplastic leukemoid reaction (PLR). Solid tumors have an association with PLR that has been documented in literature spanning decades [5]. PLR is usually found in 10% of solid tumors [6]. PPS with an extreme leukocytosis has been described only a few times in the literature [7-9].

This case report describes a case of a PPS that had PLR. We present the case of a male patient who had a 14-cm PPS with a WBC count of up to 120,000 cells/mm^3^, as well as our diagnostic investigation and the outcomes in his hospital course.

## Case presentation

Our patient was a 56-year-old African American male with a past medical history of schizoaffective disorder. He presented to our hospital with a three-month history of dry cough, orthopnea, and two to three days of right arm swelling. He also reported intermittent chest pain and progressive shortness of breath, as well as 10 pounds of weight loss in the past month. He denied hemoptysis and gastrointestinal symptoms. The patient had no consistent medical care. He had 10 pack-year smoking history. There was no known family history of cancer.

On admission, the patient was normotensive with a blood pressure of 130/66 mmHg, slightly tachycardic with a heart rate (HR) of 101 beats per minute, and slightly tachypneic with a respiratory rate (RR) of 20 breaths per minute; his oxygen saturation was 99% on room air and temperature was 37.4 degree Celsius. Physical examination revealed a pale, cachectic patient with prominent superficial vessels on the right chest. He had 2+ pitting edema of his right upper extremity as well as right axillary lymphadenopathy without cervical or supraclavicular lymphadenopathy. He did not have organomegaly. Pulmonary auscultation revealed decreased breath sounds in the right upper lobe with diffuse bilateral rales. No ptosis, miosis, or anhidrosis suggestive of Horner's syndrome was found on examination.

Blood tests revealed extreme leukocytosis (WBC of 106,500 cells/mm^3^ [reference range: 4,000-11,000 cells/mm^3^]), anemia (hemoglobin of 4.5 g/dL [reference range: 12-16 g/dL]), microcytosis (mean corpuscular volume [MCV] of 77 fL [reference range: 80-100 fL]), abnormal red blood cell count (2.64 million cell/mm^3^ [reference range: 4.5-6 million cell/mm^3^]), hypochromia (mean corpuscular hemoglobin [MCH] of 24.2 pg [reference range: 27-33 pg] and mean corpuscular hemoglobin concentration [MCHC] of 30.3% [reference range: 31-34%]), red cell distribution width (RDW) of 18.1% (reference range: 12.2%-16.1%), and platelet count of 264,000/mm^3^ (reference range: 150,000-450,000/mm^3^). Absolute neutrophil count (ANC) was 98,000 cells/mm^3^ (92%) and bands 6% (normal). Haptoglobin was 595 mg/dL (reference range: 45-215 mg/dL), lactate dehydrogenase (LDH) was 556 units/liter (reference range: 140-270 units/liter), reticulocytes was 49,400/mm^3^ (reference range: 25,000-100,000/mm^3^), reticulocyte percent was 1.7% (reference range: 0.5-2.0%), total bilirubin was 0.51 mg/dL (reference range: <1.5 mg/dL), and indirect bilirubin was 0.37 mg/dL (reference range: < 0.8 mg/dL). Prothrombin time (PT) was 11.9 seconds (reference range: 9.5-12 seconds), activated partial thromboplastin time (aPTT) was 37.9 seconds (23-33 seconds), and international normalized ratio (INR) was 1.16 (reference range: 0.9-1.1).

Peripheral blood smear showed microcytosis, neutrophilia, occasional pencil cells, and no leukoerythroblastosis, targeting, or obvious inclusion bodies.

**Figure 1:**
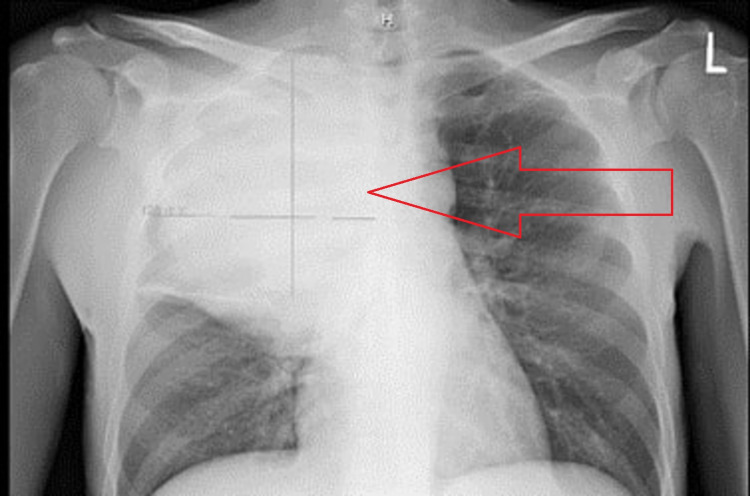
CXR shows a uniform opaque mass occupying the right upper hemithorax of around 16 x 13 cm in size. CXR, chest X-ray

Chest X-ray (CXR) showed a uniform opaque mass occupying the right upper hemithorax of around 16 x 13 cm in size (Figure 1).

**Figure 2:**
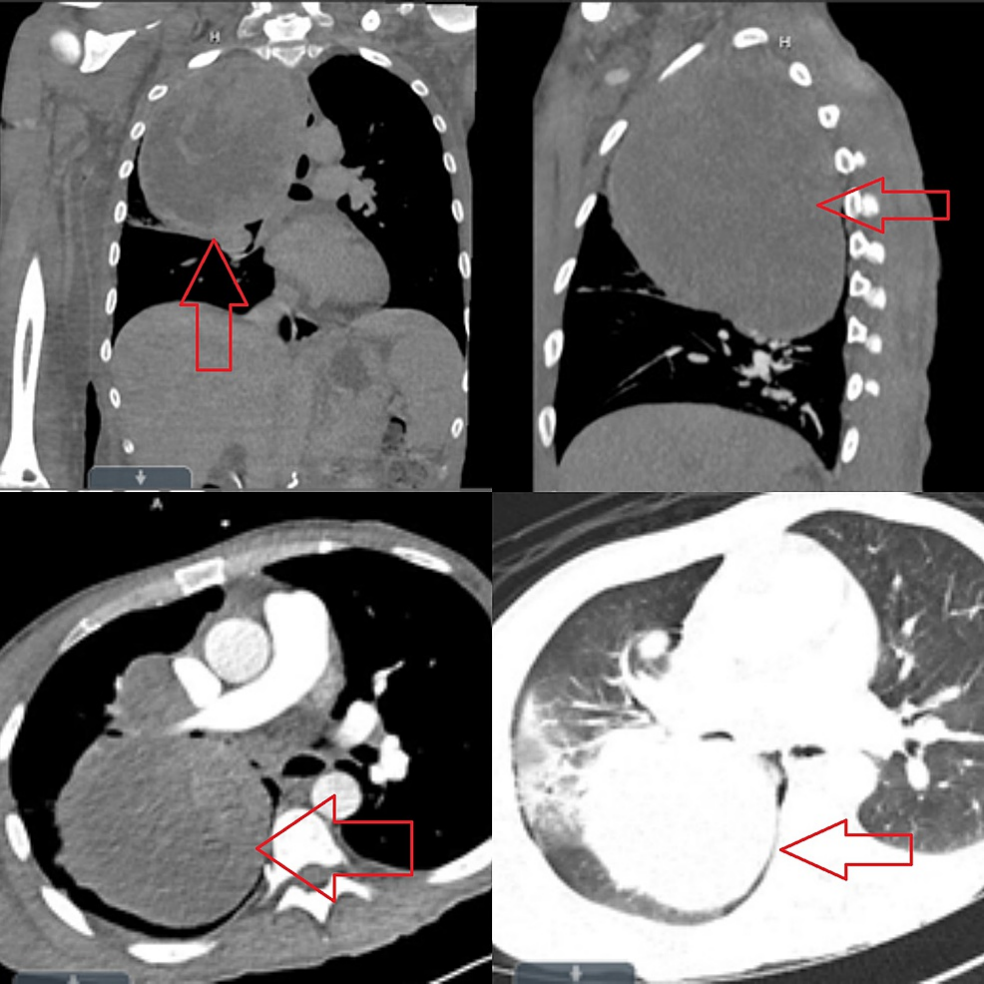
CT scan of the thorax with contrast. An irregular, thick-walled, 14-cm lung mass occupying the middle and upper hemithorax and compressing the right mainstem bronchus. The image also shows a filling defect in the left atrium, right atrial appendage, and right pulmonary vein. CT, computed tomography

Computed tomography (CT) scan of the thorax with contrast showed an irregular, thick-walled, 14-cm lung mass occupying the middle and upper hemithorax and compressing the right mainstem bronchus. It also showed a filling defect in the left atrium, right atrial appendage, and right pulmonary vein. The defect was suspicious for a thrombus or tumor invasion (Figure 2).

**Figure 3:**
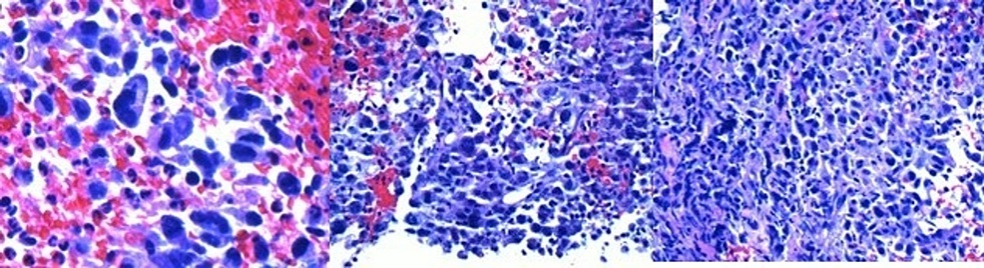
Histopathology of the mass showing a high-grade malignant tumor suggestive of sarcoma.

CT-guided biopsy of the mass showed a high-grade malignant tumor. Immunohistochemistry showed tumor cells that were positive for vimentin, and negative for keratins (Cam 5.2, Ae1/Ae3), TTF-1, synaptophysin, chromogranin, p40, LCA, desmin, and actin. Additional immunohistochemical stains were performed to classify the tumor further, which included CD34, S100, CD31, CD45, CD20, and CD3. The tumor cells were negative for all those markers, which ruled out possible anaplastic B+ and T+ T-cell lymphomas, malignant peripheral nerve sheath tumors, and vascular tumors. The conclusion of these tumor marker results was most consistent with a primary lung sarcoma (Figure 3).

Differential diagnoses of the leukemoid reaction included infection and hematological malignancy. The detailed history and physical examination were not suggestive of any infection. The respiratory and blood cultures were negative. Fungal sources were also ruled out by beta-d-glucan level of <31 pg/mL (reference range: <59 pg/mL). CXR and CT scan of the thorax did not show any pneumonia (Figures 1, 2).

The likelihood of hematological malignancies was not high because a peripheral blood smear showed only neutrophilia and occasional pencil cells. Bone marrow biopsy was not performed because sarcomas rarely involve the bone marrow [10].

CT scans of the head, neck, and abdomen/pelvis were negative for any other possible primary malignancies.

**Figure 4:**
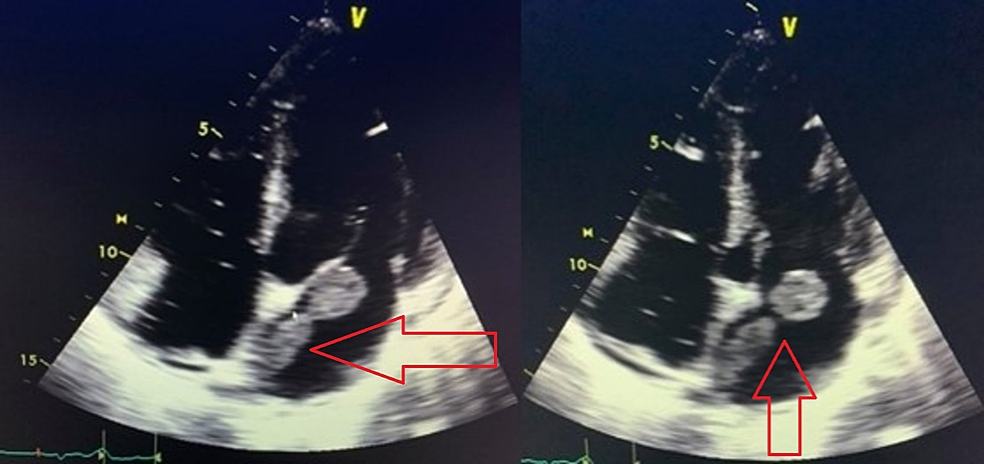
Transesophageal echocardiogram showing a large and elongated mass of 6 x 2 cm that stretched from the right upper pulmonary vein into the left atrium, suggestive of heart invasion.

Transthoracic echocardiogram showed a 1.8 x 1.7 cm globular and mobile density in the left atrium that appeared attached to the interatrial septum. Transesophageal echocardiogram showed a large, elongated, and mobile mass without a stalk that stretched from the right upper pulmonary vein into the left atrium. It measured at least 6 cm in length and 2 cm in width, suggesting heart invasion (Figure 4).

The patient was admitted to the medical intensive care unit for further evaluation and treatment. Initially, the patient was maintaining his own oxygen saturation without support. Seven days into his hospital visit, he started to require supplemental oxygen through a nasal cannula. Later, he became tachypneic with an RR of 42 breaths per minute and tachycardic with an HR of 120s with oxygen saturation decline. He was then intubated and required mechanical ventilation.

A multidisciplinary team was involved in the patient’s care. After talking to palliative medicine specialists, the family decided to change the patient’s condition to comfort measures and to withdraw life support, after which the patient passed away.

## Discussion

Primary lung sarcomas are a rare subtype of pulmonary malignancies that infiltrate the lung parenchyma. They usually form well demarcated and nonencapsulated tumors that can spread inside bronchi but rarely infiltrate bronchial epithelium [2]. Thorough clinical history, radiographic evaluation, and tissue biopsy are important in lung sarcomas’ evaluation, as it is mandatory to rule out the possibility of an alternate primary sarcoma because the lung is one of the favored metastatic sites for soft tissue sarcomas [11].

There is a strong association between lung malignancies and leukocytosis as it was concluded by many studies, such as the study by Granger et al. that evaluated 3,770 solid tumor patients; a total of 3,770 consecutive solid tumor patients with a WBC count of >40,000/microliter were retrospectively identified over a three-year period. A total of 758 (20%) patients with solid tumors and extreme leukocytosis were identified. The study found that 77 patients (about 10% of the 758 patients with extreme leukocytosis) had PLR. The etiology of the leukocytosis was hematopoietic growth factors in 522 (69%) of the 758 patients. Fewer causes of leukocytosis were infections, high-dose corticosteroids, and newly diagnosed leukemia. They concluded that approximately about 53% (41/77) had tumors with evidence of lung involvement, 17% (13/77) had non-small cell lung cancer, and 14% had soft tissue sarcomas (11/77) with none of them documented as PPS [7].

Another study that was conducted by Kasuga et al. included 227 patients with lung carcinoma; it identified 33 patients with PLR (14.5% of the study’s sample). The authors also noted that 16 patients had high serum granulocyte colony-stimulating factor (G-CSF) levels, with 12 tumors staining positively for G-CSF by immunohistochemistry [8].

A correlation between PLR and elevated serum concentrations of the hematopoietic growth factors (G-CSF), granulocyte-monocyte (GM)-CSF, and interleukin-6 in patients with lung cancer was first reported in 1977 by Asano et al. [12]. Further studies suggested a mechanism of an autocrine positive feedback loop, in which cytokines led to auto-stimulation of their respective aberrantly expressed receptors on tumor cells, resulting in tumor proliferation as well as off-target stimulation of granulocytopoiesis and corresponding PLR [6,13]. In one case report of an undifferentiated lung sarcoma in a 54-year-old female with a WBC count of 130,000 cells/mm^3^ (96% of neutrophils), the authors reported that G-CSF serum concentration was significantly elevated (6,538 pg/mL) compared to serum levels of normal controls and patients with elevated leukocytes (31 and 387 pg/mL, respectively) [14].

Unfortunately, PLR is considered a poor prognostic sign in lung sarcoma. In the study conducted by Granger and Kontoyiannis, the authors concluded that 76% of patients diagnosed with PLR died within 12 weeks [7]. In general, lung sarcoma itself carries a poor prognosis with the cumulative five-year overall survival of 27%-38% for PPS according to two different studies [2,15].

## Conclusions

Malignancies involving the pulmonary system are common. Lung carcinoma is the most common type whereas primary lung sarcoma is very rare. Because carcinomas are more common than sarcomas, they usually have more association with paraneoplastic syndromes including PLR. Our patient was a young male who was diagnosed with a PPS complicated by a PLR, which encouraged us to report to increase the healthcare professionals' awareness regarding such rare encounters.

## References

[1] Hutchinson BD, Shroff GS, Truong MT, Ko JP (2019). Spectrum of lung adenocarcinoma. Semin Ultrasound CT MR.

[2] Gołota J, Osowiecka K, Orłowski T (2018). Primary pulmonary sarcoma - long-term treatment outcomes and prognostic factors. Kardiochir Torakochirurgia Pol.

[3] Martini N, Hajdu SI, Beattie EJ Jr (1971). Primary sarcoma of the lung. J Thorac Cardiovasc Surg.

[4] Guccion JG, Rosen SH (1972). Bronchopulmonary leiomyosarcoma and fibrosarcoma. A study of 32 cases and review of the literature. Cancer.

[5] Chakraborty S, Keenportz B, Woodward S, Anderson J, Colan D (2015). Paraneoplastic leukemoid reaction in solid tumors. Am J Clin Oncol.

[6] Recio Boiles A, Lander EM, Watts GS, Nawrocki ST, Yeager AM (2018). Paraneoplastic leukemoid reaction associated with increased levels of and tumor overexpression of receptors for G-CSF, GM-CSF, and IL-6: a Clinico-Pathological-Molecular study. Blood.

[7] Granger JM, Kontoyiannis DP (2009). Etiology and outcome of extreme leukocytosis in 758 nonhematologic cancer patients: a retrospective, single-institution study. Cancer.

[8] Kasuga I, Makino S, Kiyokawa H, Katoh H, Ebihara Y, Ohyashiki K (2001). Tumor-related leukocytosis is linked with poor prognosis in patients with lung carcinoma. Cancer.

[9] Nascimento AG, Unni KK, Bernatz PE (1982). Sarcomas of the lung. Mayo Clin Proc.

[10] Bramwell VH, Littley MB, Chang J, Crowther D (1982). Bone marrow involvement in adult soft tissue sarcomas. Eur J Cancer Clin Oncol.

[11] Suster S (1995). Primary sarcomas of the lung. Semin Diagn Pathol.

[12] Asano S, Urabe A, Okabe T, Sato N, Kondo Y (1977). Demonstration of granulopoietic factor(s) in the plasma of nude mice transplanted with a human lung cancer and in the tumor tissue. Blood.

[13] Riesenberg H, Müller F, Görner M (2012). Leukemoid reaction in a patient with adenocarcinoma of the lung: a case report. J Med Case Rep.

[14] Jardin F, Vasse M, Debled M (2005). Intense paraneoplastic neutrophilic leukemoid reaction related to a G-CSF-secreting lung sarcoma. Am J Hematol.

[15] Hammar SP, Dacic S (2011). Immunohistology of lung and pleural neoplasms. Diagnostic Immunohistochemistry.

